# Supplementary material for: Correlations of amide proton transfer-weighted MRI of cerebral infarction with clinico-radiological findings
Source: PLoS One. 2020 Aug 13;15(8):e0237358. doi: 10.1371/journal.pone.0237358 (PMC7425944; doi:10.1371/journal.pone.0237358)
Supplement: S1 Appendix — (DOCX) [file pone.0237358.s001.docx]

# S1 Appendix

## Materials and Methods

### Participants

Eighteen patients with pre-stroke mRS scores of zero or one (median age, 70 years; interquartile range [IQR], 52.5 to 73.3 years) including 11 males (median age, 65 years; IQR, 43.0 to 73.0) and 7 females (median age, 70.0 years; IQR, 55.0 to 74.0) were evaluated.

### Image evaluation

The analysis was performed by a board-certified neuroradiologist (D.M., 5 years of experience) as follows. A region-of-interest (ROI) was manually drawn to include an entire hyperintense lesion on DWI, then this ROI was copied and pasted onto the amide proton transfer-weighted (APTW) image. We obtained the 10th, 25th, 50th, 75th, and 90th percentiles of the APTW signal (%) within the ROI (APT_10_, APT_25_, APT_50_, APT_75_, and APT_90_, respectively). The lowest 10% of the signal within an ROI was found below APT_10_, and the highest 10% was found above APT_90_.

Lesion sizes were measured as the maximum diameter of the infarction on the transverse diffusion-weighted images (DWI).

### Statistical analysis

Demographic and baseline clinical characteristics and the percentiles of APTW signal within the infarction were compared between the poor prognosis group (mRS score ≥2) and the good prognosis group (mRS score <2) using the Chi-squared and Mann–Whitney U tests. The correlations between the percentiles of the APTW signal within the infarction and mRS score were evaluated using Spearman’s rank correlation coefficient.

The statistical analyses were performed using JMP Pro 14.2.0 (SAS Institute, Cary, NC) under supervision of a statistician (J.K.). A p <0.05 was considered statistically significant.

## Results

The infarction etiologies were as follows: cardioembolic stroke, n = 7; non-cardioembolic stroke, n = 11 (large-artery atherosclerosis, n = 3; other determined etiology, n = 7; undetermined etiology, n = 1). Seventeen patients had infarctions in the cerebral hemispheres and one patient had infarctions in the cerebellar hemispheres. The time after onset was 54.9 hours [12.6 to 128.8] (median [IQR]) (hyperacute infarction, 1.5–15.3 hours after onset, n = 7; acute infarction, 37.8–129.3 hours after onset, n = 8; subacute infarction, 191.4–235.4 hours after onset, n = 3). The pre-stroke mRS score was 0 [0 to 1.0]. The NIHSS score was 3.0 [1.8 to 5.5]. The mRS score was 2.0 [IQR, 0 to 4.0] (good prognosis, 0–1, n = 6; poor prognosis, 2–6, n = 12). The follow-up period was 90.0 [87.8 to 91.3]. The lesion size was 47.0 mm [19.8 to 70.8] (17–24 mm, n = 5; 25–49 mm, n = 5; 50–74 mm, n = 7; 100–132 mm, n = 1).

S1 Table shows the baseline clinical characteristics and percentiles of the two groups divided according to their mRS scores at the chronic period. The baseline clinical characteristics were not significantly different between the poor and good prognosis groups. The APT_50_, APT_75_ and APT_90_ of the infarction were significantly lower in the poor prognosis group than those in the good prognosis group (median APT_50_, –0.74 [IQR, –1.42 to –0.35] vs. 0 [–0.44 to 0.42] %, p = 0.0169; APT_75_, –0.36 [–0.82 to –0.07] vs. 0.45 [–0.06 to 1.44] %, p = 0.0131; APT_90_, 0.03 [–0.44 to 0.49] vs. 0.94 [0.21 to 2.21] %, p = 0.0169).

##### **S1 Table. Demographic and baseline clinical characteristics and percentiles of APTW images of the poor prognosis group with mRS score ≥ 2 (n = 12) and good prognosis group with mRS score < 2 (n = 6).**

|  | **Poor prognosis** | **Good prognosis** | **p-value** |
| --- | --- | --- | --- |
| Gender, male:female | 7 : 5 | 4 : 2 | 0.7397 |
| Age, yrs | 72.0 (60.5 to 75.5) | 58.5 (43.3 to 70.5) | 0.1108 |
| Time after onset, hrs | 26.6 (8.1 to 83.5) | 100.8 (53.8 to 202.4) | 0.1221 |
| Lesion size, mm | 52.0 (24.6 to 73.0) | 37.0 (19.5 to 55.8) | 0.3252 |
| Pre-stroke mRS score | 0 (0 to 1.0) | 0 (0 to 1.0) | 0.9999 |
| NIHSS score | 3.0 (2.3 to 7.8) | 1.5 (0.8 to 4.0) | 0.2107 |
| Cardioembolic:Non-cardioembolic | 3 : 9 | 4 : 2 | 0.0967 |
| APT_10_, % | –1.53 (–2.00 to –0.88) | –1.07 (–1.64 to –0.25) | 0.1466 |
| APT_25_, % | –1.22 (–1.74 to –0.64) | –0.59 (–1.05 to –0.07) | 0.0549 |
| APT_50_, % | –0.74 (–1.42 to –0.35) | 0 (–0.44 to 0.42) | 0.0169* |
| APT_75_, % | –0.36 (–0.82 to –0.07) | 0.45 (–0.06 to 1.44) | 0.0131* |
| APT_90_, % | 0.03 (–0.44 to 0.49) | 0.94 (0.21 to 2.21) | 0.0169* |

##### Data are expressed as median (interquartile range). APT10, APT25, APT50, APT75, and APT90 correspond to the 10th, 25th, 50th, 75th, and 90th percentiles of APTW signal within ROI on the infarction, respectively. * indicates statistically significant (p <0.05). APTW, amide proton transfer-weighted; mRS, modified Rankin Scale; NIHSS, National Institutes of Health Stroke Scale; ROI, region-of-interest

The correlations between APTW signal within the infarction and mRS score are summarized in S2 Table. APT_50_, APT_75_, and APT_90_ were inversely correlated with the mRS score at the chronic period (APT_50_, r = –0.54, p = 0.0211; APT_75_, r = –0.59, p = 0.0099; APT_90_, r = –0.57, p = 0.0130).

##### **S2 Table. Correlations between percentiles of APTW images and mRS score (n = 18).**

|  | **mRS score** |
| --- | --- |
| APT_10_, % | r = –0.37, p = 0.1468 |
| APT_25_, % | r = –0.44, p = 0.0684 |
| APT_50_, % | r = –0.54, p = 0.0211* |
| APT_75_, % | r = –0.59, p = 0.0099* |
| APT_90_, % | r = –0.57, p = 0.0130* |

##### APT_10_, APT_25_, APT_50_, APT_75_, and APT_90_ correspond to the 10th, 25th, 50th, 75th, and 90th percentiles of the APTW signal within the infarction ROI, respectively. * indicates statistically significant (p <0.05). APTW, amide proton transfer-weighted; mRS, modified Rankin Scale; ROI, region-of-interest
