# Supplementary material for: Correlations of amide proton transfer-weighted MRI of cerebral infarction with clinico-radiological findings
Source: PLoS One. 2020 Aug 13;15(8):e0237358. doi: 10.1371/journal.pone.0237358 (PMC7425944; doi:10.1371/journal.pone.0237358)
Supplement: Attachment — Submitted filename: Response to Reviewers.docx [file pone.0237358.s003.docx]

Reviewer #2:

Reference 11 was cited at the wrong place. Currently it is cited in the introduction " or to detect subtle inflammatory changes at an early stage of multiple sclerosis [11]." This is not proper. Reference 11 not only examined APT signal, but also CEST signals at other offsets. Importantly, it applied a distribution-based analysis for CEST analysis. And in this paper the parameters APT10,APT15, ... in fact is also a quantitative desciption of APTw images. Therefore, the old reference 11 should be cited in the method within the "Quantitative analysis of APTW images" part

**RESPONSE:** Thank you for pointing this out. We have removed the reference from the introduction and have cited it in the “Quantitative analysis of APTW images” part, as a previous instance applying a histogram-based analysis on CEST data (p. 9–10, lines 181–186).
